# Stereotactic radiosurgery for pineal gland metastases: results from the international radiosurgery research foundation

**DOI:** 10.1007/s11060-026-05432-7

**Published:** 2026-01-29

**Authors:** Trent Kite, Abigail Mckenna, Rodney E. Wegner, John Herbst, Stephen Karlovits, David John, David J. Cote, Gabriel Zada, Chris Z. Wei, Suchet Taori, Neha Shastri, Cheng-Chia Lee, Hua-Che Yang, Georgios Mantziaris, Salem M. Tos, Carolina Gesteira Benjamin, Alessandro De Bonis, Gustavo Passos, Anshul Ratnaparkhi, Cameron Rivera, Türker Kılıç, Deniz Kılıç, Ali Haluk Düzkalir, Selcuk Peker, David Mathieu, Jason P. Sheehan, Ajay Niranjan, L. Dade Lunsford, Matthew J. Shepard

**Affiliations:** 1 Department of Neurosurgery, Allegheny Health Network Neuroscience Institute, 320 E North Ave Suite 208, Pittsburgh, PA 15212 USA; 2 Department of Neurosurgery, Bahçeşehir University, Istanbul, TR Turkey; 3 Department of Neurosurgery, Koc University Hospital Istanbul, Istanbul, TR Turkey; 4 Department of Neurosurgery, Université de Sherbrooke, Centre de recherche du CHUS, Quebec, CN USA; 5 Division of Radiation Oncology, Allegheny Health Network Cancer Institute, Pittsburgh, PA USA; 6 Division of Oncology, Allegheny Health Network Cancer Institute, Pittsburgh, PA USA; 7 Department of Neurosurgery, University of Southern California Keck School of Medicine, Los Angelos, CA USA; 8 Department of Neurological Surgery, University of Pittsburgh Medical Center, Pittsburgh, PA USA; 9 Department of Neurosurgery, Neurological Institute Taipei Veteran General Hospital, Taipei, Turkey; 10 Department of Neurological Surgery, University of Virgina, Charlottesville, VA USA; 11 Department of Neurological Surgery, University of Miami, Miami, FL USA; 12 Department of Neurosurgery, İstinye University, Istanbul, TR Turkey

**Keywords:** Pineal gland, Brain metastases, Stereotactic radiosurgery, Radiosurgery

## Abstract

**Purpose:**

Metastases to the pineal gland are rare. Surgical excision can be associated with high rates of morbidity. Alternatively, stereotactic radiosurgery (SRS) for brain metastases in the pineal region has not been vigorously studied.

**Methods:**

We performed a multi-institutional retrospective study for patients treated with SRS for pineal region metastases at treatment centers that comprise the International Radiosurgery Research Foundation (IRRF). Demographics, tumor characteristics, treatment parameters, and clinical outcomes were collected. The primary endpoint was local tumor control (LC). Secondary endpoints included: Overall survival (OS), distant tumor control (DC), and adverse radiation events (AREs). Kaplan-Meier and Cox regression analyses were performed to evaluate time to event endpoints and prognostic factors respectively.

**Results:**

Twenty-six patients (16 female, 62% median age 60 years (range: 16–87) with 26 pineal metastases were managed with SRS. Primary tumor histology was lung (46.2%), breast (26.9%), melanoma (7.7%), and other (19.2%). SRS treatment was up-front in the majority of cases (61.5%), adjuvant (19.2%) or salvage therapy (19.2%). The median prescription dose was 18 Gy in a single fraction. Median follow-up was 9 months (range 3–101). LC at 3-,6-,12-, and 24-months was 100%, 94.4%, 87.7%, and 87.7% respectively. DC at the same intervals were 79.2%, 73.9%, 52.8%, and 35.2%. Median OS was 32 months (range: 6–52). No evaluated prognostic factors were significantly associated with LC, DC, or OS. Among the 13 patients with symptoms related to their pineal tumor at baseline, 7 improved and 6 remained stable following SRS. Leptomeningeal spread occurred in 7.7% of patients and no cases of post-SRS hydrocephalus were observed. Overall AREs occurred in 14 (53.8%) patients, with a median time to onset of 4 months.

**Conclusion:**

SRS offers excellent local tumor control of pineal metastases with high rates of symptomatic improvement, minimal leptomeningeal spread, and limited post-SRS complications. Despite this, distant tumor control is limited in this setting and may be improved by improved systemic disease management.

## Introduction

While rare, metastatic disease to the pineal gland is often associated with a poor prognosis [1, 2]. Given its rarity, the nature and treatment associated outcomes of pineal gland metastases remain ill-defined [1]. Multimodal therapy consisting of surgery, radiation, and chemotherapy is typically utilized in this setting [1, 3]. Surgical approaches are complicated by anatomical constraints as well as the relative vascularity of these tumors which increase the risk of significant intra-operative bleeding [1, 4, 5]. Furthermore, conventional external beam radiation therapy (EBRT) poses significant risk of adverse radiation to surrounding structures [1]. Finally, chemotherapy is limited by CNS drug penetration.

Stereotactic radiosurgery (SRS) is a highly conformal and selective radiation modality that has become the standard treatment modality for patients with asymptomatic brain metastasis (BM) [6–13]. Furthermore, post-operative adjuvant SRS has been established as the standard of care for patients who require surgical excision of a supratentorial or infratentorial BM [6]. Managing pineal metastases with SRS may provide an alternative approach in settings of unfavorable tumor resection or residual/recurrent disease. Previous literature on this topic is sparse and limited to small series [14].

Leveraging the clinical experience of high-volume radiosurgical treatment centers via the International Radiosurgery Research Foundation (IRRF), we sought to describe the radiographic and clinical outcomes of patients undergoing SRS for the management of pineal gland metastases. Furthermore, we classified patients by treatment setting to elucidate settings in which SRS may confer distinct advantages.

## Methods

### Inclusion criteria

A multi-institutional retrospective cohort study was conducted utilizing data extracted from centers participating in the IRRF. Patients with metastatic tumors in the pineal gland undergoing SRS as a component of their management were selected for review. Tumor identification was based on histopathologic confirmation when SRS was employed in the post-operative, adjuvant setting. In instances where histopathology was not available, the presumed diagnosis of a pineal region BM was generally informed by the presence of other intracranial metastatic disease or the appearance of a new pineal region tumor over time in a patient with active intracranial BMs. All patients had pathologically proven extracranial cancer. Patients with leptomeningeal disease at the time of SRS or those with less than 3 months of clinical/radiographic follow up were excluded from the analysis. The Strengthening the Reporting of Observational Studies in Epidemiology checklist was used to prepare this study [15].

We further defined the SRS treatment according to one of three clinical scenarios: upfront (SRS during initial treatment with or without systemic therapy), adjuvant (SRS to residual enhancing disease or cavity following surgical resection or biopsy), salvage (SRS to recurrent disease following surgical resection, chemotherapy, or whole brain radiotherapy (WBRT).

### Data collection

Baseline demographics, therapeutic details, tumor and radiosurgical parameters, and selected clinical outcomes were collected from the electronic health record. The 5-factor modified frailty index (mFI-5) was calculated as previously described [16, 17]. Each contributing site obtained institutional review board approval for data collection. Data were then entered into a pre-designed excel sheet with removal of PHI and sent to the University of Pittsburgh for aggregation. A composite data file was then sent to the leading site’s principal investigator (M.J.S) for analysis.

### Primary endpoint

Local tumor control (LC), defined by post-SRS volumetric growth of the target volume < 20% on T1-post contrast MRI from the time of SRS, was defined as the primary endpoint of our study [18]. Local failure (local progression) occurred when post-SRS volumetric growth > 20% of the original target volume was noted on follow-up imaging.

### Secondary endpoints

Distant tumor control (DC) was defined as the absence of newly enhancing lesions beyond the index target volume on T1-post contrast MRI with distant failure (distant progression) defined as the presence of newly enhancing lesions outside of the index treatment volume [18]. Total intracranial tumor control was defined as the presence of either local ( > 20% volumetric increase in index treatment volume) or distant tumor failure (presence of newly enhancing lesions outside of the index treatment volume) at radiographic follow up. Tumor regression defined as a > 20% reduction in target volume on T1-post contrast MRI following SRS. Stable disease was defined as volumetric regression < 20% and volumetric growth < 20% of the index treatment volume following SRS. Overall survival (OS) was defined as the interval between SRS to death. Adverse radiation events (ARE) defined by the common terminology criteria (CTCAE) version 5 [19] were selected as secondary endpoints. Data were otherwise censored at the date of last clinical/radiographic follow-up.

### Statistical analysis

Continuous variables were analyzed using descriptive statistics and reported as median (interquartile range (IQR)/range). Categorical variables were analyzed in terms of frequency (%). Median OS, LC, and DC were analyzed using the Kaplan-Meier method, with patients censored on last clinical follow up for OS and last radiographic follow up for LC and DC. OS and DC were calculated on a per-patient basis, with LC calculated on a per lesion basis. Cox-regression analysis was implemented to perform univariate analysis for factors associated with local control. The proportional hazards assumption was assessed with Schoenfield residuals. All analyses were conducted with GraphPad Prism version 10.1. The threshold for statistical significance was set at *p* < 0.05.

## Results

### Cohort characteristics

A total of 26 patients with 26 metastatic tumors to the pineal gland were treated with SRS (Table 1). The median age at the time of SRS was 60 years (R: 16–87). Median performance status as determined by the Karnofsky performance scale (KPS), recursive partitioning analysis (RPA), and 5-factor modified frailty index (m-FI5) was 90 (IQR: 80–90), 2 (IQR: 1–2), and 1 (IQR: 0–1) respectively. Prior to SRS, 5 patients were diagnosed with hydrocephalus of which 4 (80%) had undergone ventriculoperitoneal shunting and one patient had undergone endoscopic third ventriculostomy (ETV). Additionally, 19 (73.1%) patients had concurrent extracranial metastatic disease. The distribution of primary tumors was lung 12 (46.2%), breast 7 (27.0%), melanoma 2 (7.7%), colorectal: 2 (7.7%), cervical 1 (3.8%), nasopharyngeal 1 (3.8%), and abdominal sarcoma 1 (3.8%). We examined patients treated with SRS in three settings: up-front 16 (61.5%), adjuvant 5 (19.2%), and salvage 5 (19.2%) (Table 1).

**Table 1:** Cohort characteristics (*N* = 26)

Median age at SRS, years (Range)	60 (16–87)
Female (%)	16 (61.5)
KPS at SRS, median (IQR)	90 (80–90)
RPA at SRS, median (IQR)	2 (1–2)
MFI-5 at SRS, median (IQR)	1 (0–1)
Symptoms and signs prior to SRS 1. Hydrocephalus (%) 2. Parinaud’s (%) 3. Ataxia (%) 4. Headache (%)	5 (19.2) 1 (3.8) 2 (7.7) 5 (19.2)
Primary tumor pathology 1. Lung (%) 2. Breast (%) 3. Melanoma (%) 4. Colorectal (%) 5. Cervical (%) 6. Nasopharyngeal (%) 7. Abdominal sarcoma (%)	12 (46.2) 7 (27.0) 2 (7.7) 2 (7.7) 1 (3.8) 1 (3.8) 1 (3.8)
SRS setting 1. Up-front (%) 2. Adjuvant (%) 3. Salvage (%)	16 (61.5) 5 (19.2) 5 (19.2)
Active extracranial disease at time of SRS (%)	19 (73.1)
Systemic therapy concurrent with SRS 1. Chemotherapy (%) 2. Targeted therapy (%) 3. Immunotherapy (%)	12 (46.2) 5 (19.2) 0 (0)
Prior therapy 1. Surgery (%) 2. Biopsy (%) 3. SRS (%) 4. WBRT (%)	2 (7.7) 3 (11.5) 2 (7.7) 3 (11.5)
CSF diversion procedure prior to SRS (%) 1. Ventriculoperitoneal shunt (%) 2. Endoscopic third ventriculostomy (%)	4 (80.0) 1 (20.0)

*Abbreviations:* KPS: Karnofsky performance status, RPA: Recursive partitioning analysis, MFI-5: Modified 5-factor frailty index, SRS: stereotactic radiosurgery, WBRT: whole brain radiotherapy, CSF: cerebrospinal fluid

### SRS parameters

The median maximum tumor diameter was 1.2 cm (R: 0.5–3.1), with a median tumor volume of 0.75 cm^3^ (R: 0.05–1.2), and a median target volume of 1.0 cm^3^ (R: 0.05–1.2) (Table 2). The tumors were treated with a median prescription dose of 18 Gy (R: 12–30) in a single fraction, to a median isodose line of 50% (R:50–94). A fractionated radiation scheme was utilized in 1 (3.8%) patient (Table 2). In the fractionated case, the target volume was treated with 30 Gy in 5 fractions.

**Table 2:** SRS parameters

Max tumor diameter (cm), median (Range)	1.2 (0.5–3.1)
Tumor volume (cm^3^), median (Range)	0.75 (0.05–1.2)
Target volume (cm^3^), median (Range)	1.0 (0.05–1.2)
Prescription dose (Gy), median (Range)	18 (12–30)
BED_10_, median (Gy)	50.4 (26.4–120.0)
Isodose line, median (%)	50 (50–94)
Fractionated (%)	1 (3.8)
Number of fractions, median (range)	1 (1–5)

Abbreviations: Gy: gray

### Tumor control

**Fig. 1:**
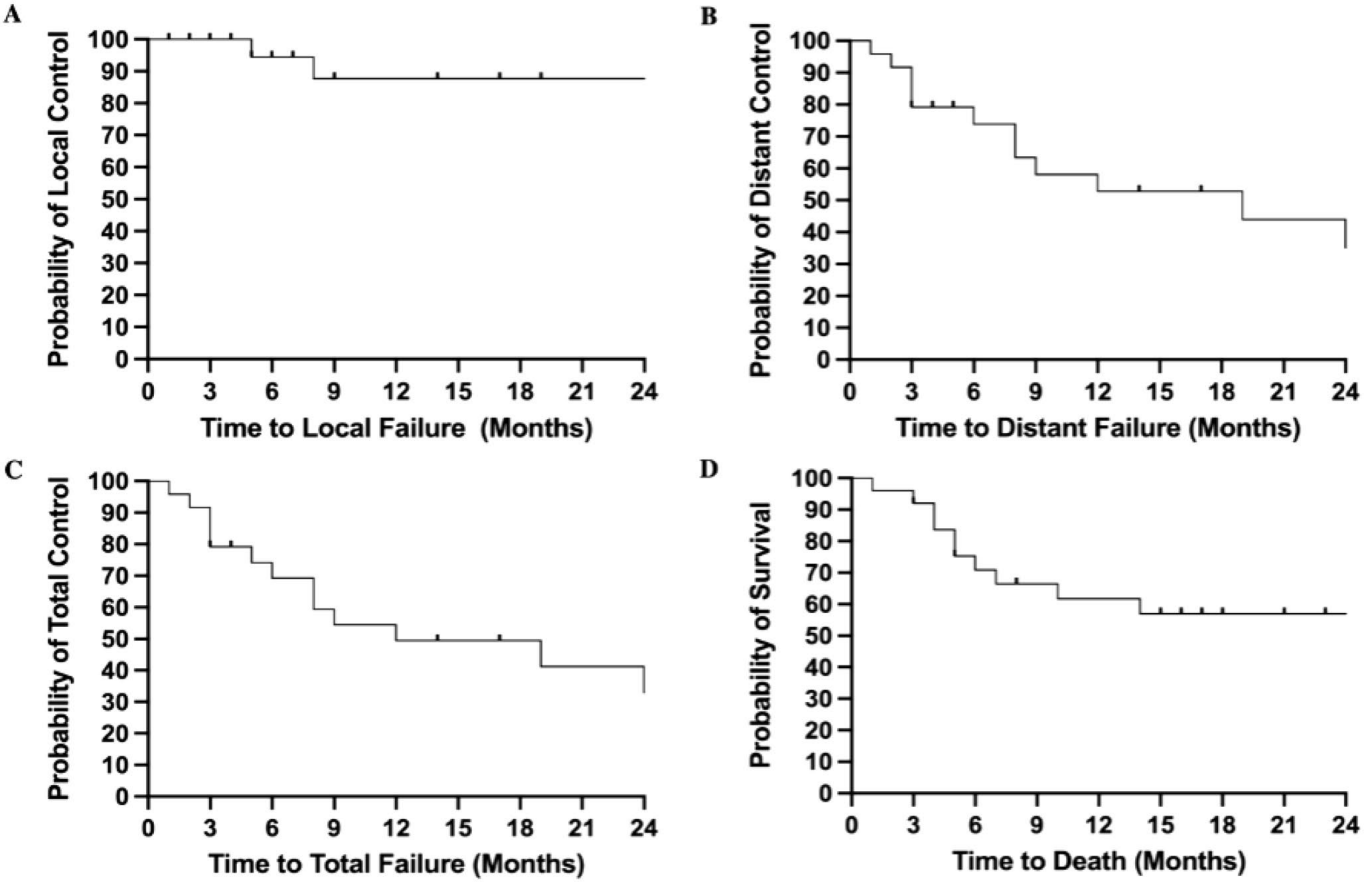
Kaplan-Meier curve of (**A**) local tumor control, (**B**) distant tumor control, and (**C**) total tumor control following SRS over 24 months of radiographic follow-up. (**D**) Kaplan-Meier curve of overall survival following SRS over 24 months of clinical follow-up

Local tumor control at 3-,6-,12-, and 24-months was 100.0%, 94.4%, 87.7%, and 87.7% respectively (Table 3 and Fig. 1A). Distant tumor control at 3-, 6-,12-, and 24-months was 79.2%, 73.9%, 52.8%, and 35.2% respectively (Fig. 1B). Total intracranial tumor control at 3-,6-, 12-, and 24-months was 79.2%, 69.3%, 49.5%, and 32.9% with a median time to total tumor failure of 12 months (Fig. 1C). While not statistically significant, several variables trended towards increased distant tumor control in the Cox regression model. These variables include controlled extracranial disease at the time of SRS (HR: 2.6, *p* = 0.16) and prior surgery or radiation (HR: 3.4, *p* = 0.18) (Table 4).

**Table 3:** Post-SRS outcomes

Adverse radiation events 1. Symptomatic (%) 2. Asymptomatic (%)	0 (0.0) 14 (53.8)
New post-SRS symptoms	3 (11.5)
Resolution of pre-SRS symptoms	7 (26.9)
LMD Development (%)	2 (7.7%)
Post-SRS hydrocephalus (%)	0 (0.0)
Further treatment 1. Surgery (%) 2. SRS (%) 3. WBRT (%)	0 (0.0) 4 (15.4) 2 (7.7)
Pineal metastasis regression (%)	15 (57.7)
Local control 1. 3-month (%) 2. 6-month (%) 3. 12-month (%) 4. 24-month (%)	100.0 94.4 87.7 87.7
Median overall survival months (95% CI)	32 (6–52)

*Abbreviations:* LMD: leptomeningeal disease

**Table 4:** Univariate predictors of distant tumor control and overall survival

Variable	HR (95% CI)	p-Value
*Distant tumor control*		
Controlled extracranial disease	2.6 (0.68–12.9)	0.16
Concurrent Systemic therapy	1.5 (0.37–5.32)	0.55
Prior surgery or radiation	3.4 (0.62–63.3)	0.18
*Overall survival*		
Age at SRS	1.0 (0.99–1.98)	0.16
Distant tumor control	2.4 (0.79–8.86)	0.12
Concurrent systemic therapy	0.87 (0.18–3.19)	0.84

Abbreviations

*P* < 0.05 statistically significant

HR > 1.0 indicates improved distant control and overall survival

### Overall survival

Overall survival at 3-,6-,12-, and 24-months was 92.0%, 70.8%, 61.7%, and 56.9% with a median of 32 months (95% CI: 6–52) (Table 3 and Fig. 1D). None of the variables tested in the Cox regression analysis demonstrated a significant association with survival. Extracranial disease control at the time of SRS trended toward improved survival (HR: 2.4, *p* = 0.12) (Table 4).

### Post-SRS outcomes

The development of new symptoms following SRS was noted in 3 (11.5%), with 6 (23.1%) experiencing resolution of pre-SRS symptoms at a median of 1 month (R:0–11) following SRS. Tumor regression was noted in 15 (57.7%) (Table 3). Leptomeningeal disease was noted in 2 (7.7%) patients. Additional surgery, SRS, and WBRT were documented in 0 (0.0%), 4 (15.4%), and 2 (7.7%) patients, respectively. Following SRS, there were 14 (53.8%) AREs (Table 3). All AREs resolved without surgical intervention. The median time to AREs was 4 months (R: 1–38).

## Discussion

### Summary of findings

Stereotactic radiosurgery for pineal metastases provides a promising long term LC rate of 87.7% at 24-months. Additionally, following SRS we found no reported instances of symptomatic adverse radiation events. Furthermore, > 25% of the cohort experienced resolution of their tumor associated symptoms after SRS. The combination of high local control rates, low symptomatic adverse radiation events, and frequency of pre-SRS tumor symptom resolution supports the efficacy of SRS as a therapeutic modality in this patient population. Unfortunately, total tumor control was discouraging and is primarily attributable to a low rate of distant tumor control (35.2% at 24-months). Extracranial disease control and concurrent systemic therapy at the time of SRS demonstrated a trend toward improved distant control, albeit failing to reach statistical significance. Interestingly, we reported an association between distant tumor control and overall survival. This implies that SRS for pineal metastases may be an efficacious therapy; however appropriate patient selection is critical for long term outcomes. Additionally, comparative analysis with alternative radiation modalities, namely WBRT, would further improve the understanding of SRS in this setting.

### Tumor control

Overall, our cohort demonstrated excellent LC (87.7%), with only two tumors fulfilling criteria for local failure. Indeed, greater than half of the cohort underwent tumor regression following SRS. Consistent with contemporary literature, distant tumor failure accounts for the reduction in total tumor control [19–21]. One plausible explanation for high rates of distant tumor failure is the presence of uncontrolled extracranial disease at the time of SRS. Our cohort demonstrated an association between extracranial disease control and distant tumor control, albeit not statistically significant. This may be related to the receipt of systemic chemotherapy at the time of SRS, as this was also associated with distant tumor control, however without statistical significance. While the aforementioned points are speculative in nature, given the sample size of the cohort it is reasonable to attribute the lack of statistical significance partly to limited power. These trends highlight the importance of appropriate patient selection, especially in relapsed/refractory disease states. Additionally, the presence of microscopic disease undetected on pre-treatment imaging may exist at the time of SRS. This conundrum is a limitation of SRS, whereby the highly conformal nature creates high target selectivity at the omission of potentially treating undetectable disease. Therefore, a theoretical advantage of WBRT in the setting of intracranial metastases is the distribution of radiation to undetectable disease. Consequently, one may view distant failure as an expected outcome in the setting of SRS, while WBRT may be advantageous in this respect. Interestingly, a 2025 meta-analysis demonstrated no significant difference in distant control rates across 5 studies comparing WBRT versus SRS (RR: 0.83, *p* = 0.41) [22]. However, these results were derived from a limited number of studies reporting on a heterogenous cohort of primary tumor histologies and included only one randomized controlled trials (RCTs) [22].

### Survival

Survival in our cohort compares favorably to that of other CNS metastases treated with SRS and surgical cohorts of pineal metastases. Palmisciano et al. [1] Reported on a cohort of 13 surgically resected pineal metastases demonstrating an OS of 3 months with actuarial rates of 41, 31, and 24% at 3-, 6-, and 12-months respectively [1]. Current meta-level data estimates of OS following SRS for a CNS metastasis is estimated at a median of 12.2 months (95% CI: 11.0–13.4 months) [21]. In a retrospective-cohort study examining 25 patients with a variety of pineal histologies, 5-year survival was reported at 60% [23]. With respect to WBRT outcomes, Alrasheed et al. demonstrated a survival benefit with a mean difference of 4.05 months (*p* < 0.001) when comparing SRS to WBRT [22]. When exclusively evaluating RCTs, statistical significance was lost [22]. It is challenging to extrapolate these results, as they were derived from a mixed histology cohort. This further highlights the need for specific research evaluating WBRT and SRS outcomes in the setting of pineal gland metastases. As is true for most patients with CNS metastases, survival is a multifactorial outcome dependent on a variety of factors beyond local tumor control. LC is important to reduce tumor complications and restore physiological function. However, optimization of systemic medical therapy prior to and following SRS is necessary to prolong survival in this patient population.

### Adverse events

Because of the proximity to the ventricular system and absence of a typical blood-brain barrier, management of pineal region tumors carries an increased risk of leptomeningeal disease [1, 24, 25] Palmisciano et al. published on 47 patients with pineal metastases managed with surgical resection, WBRT, and SRS reporting a crude LMD rate of 22.2% [1]. Mechanical disruption of the tumor capsule during a biopsy or surgical resection may facilitate tumor cell seeding of the CSF. A sub-analysis of 12 patients in this cohort undergoing adjuvant SRS following biopsy, complete or partial resection demonstrated a LMD rate of 16.6% [1]. Therefore, SRS may be considered as an alternative in cases where the risk of CSF seeding is increased or as a method to sterilize a resection cavity. Additionally, there is ongoing debate regarding the timing of SRS (neoadjuvant, intra-operative, or adjuvant) as a strategy to reduce LMD. When evaluating the WBRT literature, LMD rates were favorable with WBRT for intracranial BMs (SRS vs WBRT, HR: 3.09, P: 0.003) [22]. However, concerns over WBRT related neurotoxicity have limited utilization. Within our cohort an LMD rate of 7.7% was found, both cases occurring in the setting of “salvage” treatment. Overall, slightly more than half of the cohort experienced AREs, with 100% asymptomatic, which may indicate clinical insignificance. Overall morbidity following resection of pineal tumors ranges from 18 to 56.3% [26–32].

### Strengths and limitations

This is one of the only retrospective series examining outcomes of pineal metastases treated with SRS. Utilization of a multicenter, international study design increases generalizability of the findings. Furthermore, the reporting of outcomes in accordance with widely accepted research guidelines facilitates interpretation of results across future studies. Nonetheless, our sample size is low, largely attributable to the rarity of the pathology, and the retrospective design increases the risk of selection bias. Related to the sample size, local failure event rates were too few to permit multivariate analysis. While the absence of such analysis does not diminish the importance of the work, it represents an area of future investigation. Additionally, data were collected over the span of two decades, over which time there has been rapid evolution of systemic therapy. Modern immunotherapy and targeted therapy agents may drastically alter the clinical disease course and facilitate the effects of radiosurgery. A Detailed description of the post-SRS AREs was not obtained in this study. Furthermore, a more detailed analysis of adverse radiation events and poor outcomes, particularly rates of LMD and CNS related mortality following SRS, represent a relevant area of outstanding research. Finally, direct comparison to WBRT-treated patients was not performed, which is critical to understanding the risk and benefit of potential neurocognitive toxicity balanced against differences in tumor control.

## Conclusion

This is one of the only retrospective series exclusively examining the clinical and radiographic outcomes of pineal gland metastases managed with SRS. In this study, SRS for pineal region metastases provided durable local tumor control with no symptomatic AREs. Distant tumor control limits overall total intracranial tumor control and may partially account for limited overall survival in such cases. Optimizing patient selection may be critical for extracting the maximal benefit from SRS in this setting. Future studies replicating our findings are warranted. There is also a need to more closely examine tumor control, survival, and treatment related toxicity between SRS and WBRT in the setting of pineal gland metastases.

## Data Availability

Data is available upon reasonable request to the corresponding author
